# Bullying and sexual abuse and their association with harmful behaviours, antidepressant use and health-related quality of life in adulthood: a population-based study in South Australia

**DOI:** 10.1186/s12889-018-6367-8

**Published:** 2019-01-07

**Authors:** David Alejandro González-Chica, Julio Licinio, Michael Musker, Mali Wong, Jacqueline Bowden, Phillipa Hay, Catherine Chittleborough, Nigel Stocks

**Affiliations:** 1 Discipline of General Practice, Adelaide Medical School, The University of Adelaide, Helen Mayo North, Level 1, Room N113, North Terrace Campus, Adelaide, SA 5005 Australia; 2 College of Medicine, Upstate Medical University, Syracuse, NY USA; 3 Mind & Brain Theme, South Australian Health & Medical Research Institute, Adelaide, SA Australia; 4 Population Health Research Group, South Australian Health & Medical Research Institute, Adelaide, SA Australia; 5 Translational Health Research Institute, School of Medicine, Western Sydney University, Sydney, NSW Australia; 6 School of Public Health, The University of Adelaide, Adelaide, SA Australia

**Keywords:** Child abuse, Sexual, Bullying, Behavioural symptoms, Quality of life, Mental health

## Abstract

**Background:**

Few representative sample studies have reported estimates of bullying and sexual abuse in Australia. By using face-to-face interviews and self-labelling questions, we investigated the prevalence of these forms of abuse and their relationship with current harmful behaviours (smoking dependence, excessive alcohol intake, binge eating), antidepressant use, and the physical (PCS) and mental (MCS) components of health-related quality of life.

**Methods:**

This study was a population-based survey that investigated 2873 South Australians in 2015 (48.8 ± 18.1 years; 49.3% males). Bullying and sexual abuse (age of onset and duration) and their outcomes were investigated through household interviews. Associations were adjusted for sociodemographic variables by using regression models.

**Results:**

45.6% (95% CI 43.3–47.9) of the participants were bullied, and 10.4% (95% CI 9.1–11.9) sexually abused; 7.3% (95% CI 6.2–8.5) reported experiencing both forms of abuse. Moreover, 15.8% of those bullied and 15.0% of those sexually abused suffered from these forms of abuse for > 24 months. Smoking dependence (7.8%) was twice as frequent among those who experienced bullying for > 24 months or when sexual abuse occurred in childhood (< 10 years) or adulthood (20+ years) or lasted ≥1 month. Excessive alcohol intake (14.3%) was more frequent when bullying occurred in childhood or lasted > 24 months. Binge eating (8.1%) was more frequent among those bullied or sexually abused in adulthood, but duration did not show a clear pattern. Antidepressant use was up to four times more likely, and PCS or MCS lower among those who were bullied or sexually abused, independent of when these forms of abuse started or their duration. The cumulative adverse relationship of bullying and sexual abuse with the investigated outcomes was more evident for smoking dependence, binge eating, PCS, and MCS than for antidepressant use, but no association was observed with alcohol intake.

**Conclusions:**

The use of self-labelling questions to investigate sensitive areas such as bullying and sexual abuse in a survey is feasible. Such questions provided estimates that are consistent with findings from studies using more detailed instruments. Bullying and sexual abuse have an additive adverse association with various outcomes. Identifying survivors of both forms of abuse is important to avoid more serious consequences.

**Electronic supplementary material:**

The online version of this article (10.1186/s12889-018-6367-8) contains supplementary material, which is available to authorized users.

## Background

Childhood experiences of abuse have been associated with long-term health consequences [1–6]. Childhood sexual abuse is one of the most investigated forms of maltreatment and has been identified as a modifiable risk factor for mental disorders across the life course [7]. In terms of magnitude, sexual abuse is a global problem affecting all age groups, with a prevalence as high as 59% in some low-income countries and a lifetime risk of rape (attempted or completed) of 20% in women and 4% in men [8, 9]. However, sexual abuse is also a concern in high-income economies. In the United Kingdom, it is estimated that one in every 250 women aged 16–59 years is raped every year, but only 18% of these rapes are reported to the police [10]. Although most studies have focused on the psychological consequences of rape, many of the health implications are equally applicable to victims of other forms of sexual abuse [7, 8, 10].

Other forms of childhood abuse have proven worthy of investigation. Two independent meta-analyses published in 2012 [7] and 2016 [3] showed that the long-term adverse effects of early psychological/emotional abuse (usually inflicted by adults) on depression later in life are even stronger than the effects of physical or sexual abuse. Moreover, early psychological/emotional maltreatment has also been related to drug use, suicide attempts, risky sexual behaviour, and adverse physical health outcomes (i.e., neurological, musculoskeletal and immune response disorders) [4, 6, 7, 11].

Being bullied is the most common form of psychological/emotional maltreatment by peers experienced early in life; it has an estimated prevalence of 36% among school-age children [12–14]. Bullying is defined as a harmful and aggressive behaviour by peers that is carried out repeatedly and involves an imbalance of power [12, 13]. Although perpetrators may consider it a harmless rite of passage with few long-term consequences, bullying has been associated with severe outcomes, including school absenteeism, anxiety, depression, and suicidality [14–16]. Although most research investigating the long-term health consequences of bullying has focused on school bullying, it occurs throughout the lifespan in all societies and in many workplaces [13–16].

Several authors have suggested that multiple forms of abuse are likely to co-occur and become recurrent, showing a dose-response effect on different health outcomes [3, 4, 6, 7]. A systematic review published in 2017 that included 37 high-quality studies showed that individuals who suffered four or more adverse childhood experiences (i.e., sexual, physical, emotional or other forms of abuse) were between two and seven times more likely to smoke, have problematic alcohol or drug use, be involved in risky sexual behaviour or interpersonal and self-directed violence than those with no history of childhood adversity [6].

Childhood maltreatment also seems to affect other outcomes in adulthood, such as health-related quality of life (HRQoL) [4, 17]. HRQoL is a construct that represents an individual’s perception of the impact of ill health on diverse life domains [18]. This outcome is closely related to functional capacity, adherence to medical treatments, disease severity and mortality [19, 20]. Therefore, the exploration of different outcomes related to bullying and sexual abuse in the same population may help clinicians to identify individuals with a greater vulnerability to health impairment and to provide interventions that aim to mitigate their suffering.

In Australia, Moore et al. (2015) synthesised evidence from 23 national studies and found that multiple forms of child maltreatment are highly interrelated and are associated with depression, anxiety and intentional self-harm [21]. Nonetheless, only a few studies in that review included a representative sample of Australian adults. Systematic reviews have highlighted other methodological limitations in the available literature. These limitations include a lack of population-based samples, studies involving a low percentage of males or older adults, and few studies investigating the age of onset, duration and/or severity of abuse [3, 4, 6, 21, 22].

Therefore, the main objective of this study was to investigate the frequency, age of onset and duration of bullying and sexual abuse in a representative sample of adults living in South Australia (SA). We also wanted to examine the association of these variables with harmful behaviours (smoking dependence, excessive alcohol intake, and binge eating), antidepressant use, and HRQoL. Both the independent associations of bullying or sexual abuse with adverse health outcomes and the combined effect of each form of maltreatment were explored. This study also aimed to estimate the likelihood of having ever experienced bullying and/or sexual abuse based on the number of adverse health outcomes the individual reported.

## Methods

A cross-sectional study using household interviews (Health Omnibus Survey) was performed. The main goal of the Health Omnibus Survey is to collect, analyse and interpret data that can then be used to plan, implement and monitor health programmes and other initiatives [23]. In 2015, the survey comprised 150 health questions and was administered to a representative sample of adults living in South Australia, a state with approximately 1.7 million inhabitants (73% living in metropolitan areas) [24].

Details regarding the methodology have been published elsewhere [23, 25]. In summary, a multistage sampling process was conducted between September and December 2015, when clusters of 10 residences were systematically selected from 530/3939 statistical areas (including urban and rural areas) [24]. One dweller aged 15+ years was randomly chosen (last person to have a birthday) in each household. Individuals were excluded if they were terminally ill/mentally incapacitated (*n* = 104) or unable to speak English (*n* = 87). The final sample included 3005 individuals (71.1% of the 4226 eligible participants), but only adults (20+ years) were included in the analyses (*N* = 2912).

The interviews took on average 35 min to complete (range 30–40 min), and the survey included questions on sociodemographic variables, HRQoL, self-reported height and weight, lifestyle habits and risk perceptions (i.e., physical activity, food consumption, alcohol intake, smoking), participation in preventive activities, sleeping habits, self-reported chronic health conditions (diagnosis and management, including physical and mental health diseases), falls, eating disorders, bullying, and sexual abuse.

### Bullying and sexual abuse

Due to the sensitive nature of these questions, the non-health professional researchers were given support regarding how to ask them and were advised to avoid engaging in any form of dialogue about the details of the experience. If the participant was an adolescent, these sensitive questions were not asked. Cards were provided with contact numbers for local helplines and contacts for anyone who might become distressed by such questions. All the participants were provided with the following opening statement: “*Please note these next few questions are about how experiences relate to physical health. We do not need to know specific details. The questions may be confrontational for some people, and you can choose not to answer them if you are not comfortable*.” No description of bullying or sexual abuse was given to the interviewees prior to the interview questions.

The main exposure variables were then investigated through the following self-labelling questions: *“Have you ever been bullied at school or work?”* and *“In your lifetime, have you ever experienced any type of sexual abuse?”.* After each question, the participants were asked about the age when the last event occurred and how long this form of abuse lasted. The age at the onset of each form of abuse was then calculated (i.e., if the last episode of sexual abuse occurred at 12 years, and it lasted five years, the age of onset = seven years) and classified as < 10 years (childhood), 10–19 years (adolescence), or ≥ 20 years (adulthood) [26]. To investigate a dose-response effect [5, 7, 13], the lifetime duration of bullying or sexual abuse was also classified as < 1 month, 1–24 months, or > 24 months. Finally, to investigate the cumulative effect of both bullying and sexual abuse (ever occurred, independent of duration), they were combined into one categorical variable with four categories: 1) Neither bullying nor sexual abuse; 2) just bullying; 3) just sexual abuse, or; 4) both bullying and sexual abuse.

The survey did not collect data regarding the specific form of bullying or sexual abuse the victims experienced, and no other forms of early maltreatment were investigated.

### Harmful behaviours and antidepressant use

Considering that bullying and sexual abuse have been related to behaviours that are compulsive, repetitive, distressful, and/or excessive and have deleterious physical/mental consequences [4, 5, 7, 11, 13], three binary variables (yes/no) were investigated as indications of harmful behaviours: smoking dependence, excessive alcohol intake, and binge eating. Smoking dependence was determined based on the Heaviness of Smoking Index [27], which allocates points for the average number of cigarettes smoked every day (0 = 10 or less; 1 = 11–20; 2 = 21–30; 3= > 30 cigarettes/day) and the time from waking to smoking the first cigarette of the day (0 = 60+ minutes; 1 = 31–60 min; 2 = 5–30 min; 3 = < 5 min). A score ≥ 3 points was used as the cut-off for smoking dependence. Alcohol intake was investigated using separate questions for the frequency of drinking and the number of standard drinks (using a prompt card showing the equivalence of a standard drink for different types of alcohol) consumed in the last 12 months; excessive alcohol intake was defined as 5+ standard drinks of alcohol consumed 5+ days/week [28]. Binge eating in the past three months was self-reported through the following question: “*I would like to ask you about episodes of overeating. By overeating, or binge eating, I mean eating an unusually large amount of food in one go and at the time feeling that your eating was out of control*. *Over the past 3 months, how often have you overeaten? Would you say… 1. Not at all, 2. Less than weekly, 3. Once a week, or 4. Two or more times a week.”* The level of distress reported by the participants was then investigated (“*not at all”*, “*a little*”, or “*a lot*”). Binge eating was defined as overeating of any duration associated with “a little” or “a lot” of distress.

Respondents were considered active users of antidepressants if they were currently using any of the 22 different antidepressant drugs available on the Pharmaceutical Benefits Scheme (PBS) in Australia. A list of these medications and their alternative commercial names was used during the interview (again using a prompt card).

### Health-related quality of life

HRQoL was investigated using the physical (PCS) and mental (MCS) component summary scores of the Medical Outcomes Study Short Form 12 (SF-12v1). The 12 questions in this instrument assess impairment in physical and mental health in the past four weeks. The questions were combined to generate scores; the scores had a mean value of 50 and a standard deviation of 10, with higher values indicating a better HRQoL [29, 30].

### Confounding variables

The sociodemographic variables included as possible confounders were sex (male or female), age (in years, including a quadratic term for nonlinear associations), marital status (married/living with a partner - yes or no), area of residence (urban or rural), quintiles of a macro-level socioeconomic position indicator (2011 Australian Socio-Economic Indexes for Areas Index of Relative Socioeconomic Advantage and Disadvantage, SEIFA-IRSAD), highest education level attained (bachelor’s degree or higher; trade qualification; certificate/diploma; secondary; less than secondary), working status (employed full-time; employed part-time; unemployed [including home duties and students]; retired), and dwelling type (owned, rented or community/government housing). The SEIFA-IRSAD is an index based on a range of census variables and an indicator of the relative economic and social advantage/disadvantage of people and households within an area (higher scores indicate that the respondent lives in a more advantaged area) [31].

### Data analysis

Logistic regression was used to investigate the distribution of bullying and sexual abuse according to sociodemographic variables (adjusted for sex and age), and the marginal adjusted prevalence was then estimated. Regression models were also used to investigate the association of bullying and sexual abuse with harmful behaviours, antidepressant use (logistic regression), and HRQoL (linear regression), with full adjustment for all sociodemographic variables. The results are expressed as adjusted odds ratios (ORs, for binary outcomes) or regression coefficients (βs, for PCS and MCS) with their respective 95% confidence intervals (95% CIs). When assessing the cumulative effects of bullying and sexual abuse on these outcomes, marginal adjusted prevalence or means were estimated accordingly and reported with their respective 95%CIs. Heterogeneity tests were conducted to examine the association between age and bullying and sexual abuse according to age, as well as between bullying and sexual abuse and all outcomes according to the participant’s sex and use of antidepressants; the association was considered positive when the interaction terms showed a *p*-value < 0.10 [32]. Finally, multinomial regression was used to estimate the adjusted predicted likelihood of past abuse (neither bullying nor sexual abuse, just bullying, just sexual abuse, or both bullying and sexual abuse) considering the adverse health outcomes associated with these forms of abuse as predictors (harmful behaviours, antidepressant use, and HRQoL) and was adjusted for all sociodemographic variables. The results of this prediction are presented graphically as % of each form of past abuse according to the number of adverse health outcomes within the same individual.

The statistical software STATA 14.0 (StataCorp, Texas, USA) was used for analysis, and only individuals with complete exposure variable and outcome data were included. All results were weighted to the inverse of the individual’s probability of selection within the household (re-weighted to account for the estimated resident population in SA in 2014 according to age and sex) and were analysed considering the sampling design (clusters of statistical areas) [23, 24]. Participants provided verbal rather than written informed consent, due to the practicalities of carrying out a large-scale survey and the low-risk nature of the survey content. All procedures performed in this study were approved by the University of Adelaide Human Research Ethics Committee (project H-097-2010).

## Results

Of the 2912 individuals aged 20+ years who were interviewed in the study, 1.1% (*n* = 33) refused to answer the questions on sexual abuse (*n* = 11 also refused to answer the questions on bullying), while another six participants had some missing data for other investigated outcomes. The mean age of the final sample (unweighted *N* = 2873) was 48.8 ± 18.1 years (49.3% males). The prevalence of ever being bullied was 45.6% (95% CI 43.3–47.9), the prevalence of ever being sexually abused was 10.4% (95% CI 9.1–11.9), and 7.3% (95% CI 6.2–8.5) reported both forms of abuse. Approximately 45% of all cases of bullying and a similar proportion of sexual abuse cases started in adolescence (10–19 years), while 20.2 and 32.6% started in childhood (< 10 years). Moreover, 15.8% of those who were bullied and 15.0% of those who were sexually abused suffered from these forms of abuse for more than 24 months, with a prevalence up to 3.7 times higher when the abuse started in childhood rather than later in life (Additional file 1).

Sexual abuse was 4.7 times more frequent in females than in males, while the rates of bullying were similar for both sexes (Table 1). Conversely, the prevalence of bullying decreased with age, while that of sexual abuse remained relatively stable in most age groups. However, there was evidence of an interaction between sex and age in their association with both forms of abuse (Additional file 2): the rate of bullying was similar in males and females younger than 50 years, but bullying was less frequent among elderly women; the rate of sexual abuse remained steady at approximately 4% among men of all age groups, while among women, the highest frequency was among those aged 35–64 years.

**Table 1:** Sample distribution and prevalence^a^ of bullying and sexual abuse according to sociodemographic variables among individuals aged ≥20 years in South Australia, 2015 (unweighted *N* = 2873)

Variables	%	Bullying (ever) %	Sexual abuse (ever) %
Overall		45.6	10.4
Sex			
Male	49.3	44.8	3.6***
Female	50.7	46.4	17.1
Age			
20–34 years	27.1	52.8***	9.6
35–49 years	24.7	50.8	12.1
50–64 years	26.1	47.4	12.1
65–79 years	17.0	31.8	9.3
80+ years	5.2	19.4	2.9
Marital status			
Single/divorced/widowed	33.0	49.2*	14.4***
Married/living with a partner	67.1	43.8	8.6
Residence area			
Urban	74.7	45.0	10.5
Rural	25.3	47.3	10.1
Socioeconomic position (quartiles)^b^			
High	27.8	45.1	12.2
Middle-high	22.2	41.2	9.5
Middle-low	25.0	48.9	8.8
Low	25.0	46.6	10.9
Educational level			
Bachelor or higher	25.7	48.2*	9.2***
Certificate/diploma	28.3	48.8	12.9
Trade qualification	13.3	44.2	6.7
Secondary	24.0	42.3	10.8
Less than secondary	8.7	36.9	6.9
Working status			
Employed full-time	37.4	47.1*	11.1*
Employed part-time	20.5	47.7	9.9
Not working	19.6	49.6	13.9
Retired	22.5	36.5	7.1
Dwelling			
Owned	70.2	44.4	9.1**
Rented	23.6	47.8	12.9
Community/government housing	6.1	50.0	16.1

The weighting of data can lead to rounding effects and totals that do not add up to 100%

^a^Prevalence of bullying and sexual abuse adjusted for sex and age

^b^Based on the Socio-Economic Indexes for Areas Index of Relative Socio-economic Advantage and Disadvantaged (SEIFA-IRSAD)

*p < 0.05; **p < 0.01; ***p < 0.001 (chi-square test)

Table 1 also shows that both forms of abuse were less prevalent among those who were married/living with a partner, but no difference was observed regarding residence area or socioeconomic position. Bullying rates were also higher in all groups with an educational level equal to or higher than secondary school, while sexual abuse was more common among those with a certificate/diploma. Both conditions occurred less frequently among retired individuals, while those who were not working showed the highest prevalence of sexual abuse. Although dwelling type was not associated with bullying, those living in community/government housing were 1.8 times more likely than homeowners to report a history of sexual abuse.

Table 2 presents the association between age of onset and duration of bullying or sexual abuse with smoking dependence (7.8%), excessive alcohol intake (14.3%), binge eating (8.1%) and current use of antidepressants (17.2%). Smoking dependence was not associated with the age when bullying started but was more frequent when it lasted more than 24 months, while those who suffered sexual abuse in childhood or adulthood or lasted ≥1 month were twice as likely to be smoking dependent compared to those who were never abused. On the other hand, only being bullied (starting in childhood or lasting > 24 months) was related to excessive alcohol intake. Additionally, bullying at any age or that lasted 1–24 months was associated with a higher frequency of binge eating and was more frequent when sexual abuse started in childhood or adulthood or lasted less than one month. On the other hand, the use of antidepressants was up to four times more frequent among those who were bullied or sexually abused, independent of the age of onset or duration.

**Table 2:** Adjusted association^a^ of bullying and sexual abuse with smoking, alcohol intake, binge eating and antidepressant use among individuals ≥20 years in South Australia, 2015 (unweighted *N* = 2873)

	%	Smoking dependence^b^ (7.8%)	Excessive alcohol intake^c^ (14.3%)	Binge eating^d^ (8.1%)	Antidepressant use (17.2%)
		OR (95% CI)	OR (95% CI)	OR (95% CI)	OR (95% CI)
BULLYING					
Age when started		*p* = 0.533^e^	*p* = 0.161^e^	*p* = 0.015^e^	p < 0.001^e^
Never	54.4	Ref	Ref	Ref	Ref
< 10 years	9.2	1.51 (0.82;2.77)	1.62 (1.04;2.51)	1.77 (1.00;3.21)	1.67 (1.15;2.41)
10–19 years	20.7	0.97 (0.64;1.47)	1.23 (0.90;1.69)	1.66 (1.13;2.45)	1.95 (1.47;2.60)
20+ years	15.7	1.17 (0.74;1.82)	1.09 (0.72;1.66)	1.87 (1.20;2.93)	2.03 (1.50;2.74)
Duration (months)		*p* = 0.070^f^	*p* = 0.005^f^	*p* = 0.001^f^	p < 0.001^f^
Never	54.4	Ref	Ref	Ref	Ref
< 1 month	15.2	0.87 (0.48;1.60)	1.05 (0.68;1.64)	1.45 (0.88;3.37)	1.45 (1.05;2.00)
1–24 months	23.2	1.00 (0.65;1.54)	1.35 (0.99;1.85)	2.02 (1.36;2.99)	2.07 (1.59;2.69)
> 24 months	7.2	2.18 (1.22;3.91)	1.60 (1.04;2.46)	1.56 (0.88;2.77)	2.50 (1.68;3.70)
SEXUAL ABUSE					
Age when started		*p* = 0.009^e^	*p* = 0.298^e^	*p* = 0.004^e^	p < 0.001^e^
Never	89.6	Ref	Ref	Ref	Ref
< 10 years	3.4	2.51 (1.26;4.99)	1.07 (0.54;2.14)	1.85 (1.12;3.06)	3.58 (2.22;5.78)
10–19 years	4.7	1.42 (0.82;2.47)	0.73 (0.37;1.47)	1.20 (0.67;2.14)	2.02 (1.31;3.11)
20+ years	2.4	2.62 (1.23;5.59)	0.40 (0.14;1.15)	3.36 (1.63;6.93)	2.57 (1.31;5.05)
Duration (months)		p = 0.001^f^	*p* = 0.279^f^	p = 0.009^f^	p < 0.001^f^
Never	89.6	Ref	Ref	Ref	Ref
< 1 month	5.9	1.40 (0.84;2.33)	0.82 (0.43;1.58)	2.01 (1.16;3.48)	2.24 (1.56;3.23)
1–24 months	2.9	2.74 (1.46;5.15)	0.55 (0.20;1.51)	1.57 (0.89;2.76)	2.53 (1.46;4.37)
> 24 months	1.6	3.35 (1.32;8.51)	0.91 (0.24;3.44)	1.52 (0.65;3.53)	4.53 (2.36;8.67)

OR = odds ratio; 95% CI = 95% confidence interval;

^a^Results adjusted for sex, age, marital status, area of residence, education level, working status, socioeconomic position (Socio-Economic Indexes for Areas Index of Relative Socio-economic Advantage and Disadvantaged), and type of dwelling. Bullying and sexual abuse were also mutually adjusted

^b^Heaviness of Smoking Index ≥3 points

^c^≥5 standard drinks of alcohol/day

^d^Overeating or eating an unusual large amount of food in one go (“out of control”) + distress associated with this behaviour

^e^Likelihood-ratio test for heterogeneity

^f^Likelihood-ratio test for trend

Table 3 shows the association between bullying or sexual abuse with PCS (mean = 48.4 ± 10.4 points) and MCS (mean = 52.5 ± 8.7 points). In general, bullying and sexual abuse were associated with a lower PCS and MCS, independent of the age at onset or its duration. The strongest relationships with PCS were for bullying starting in adolescence, sexual abuse starting in adulthood, or when both forms of abuse lasted 24 months or more. The magnitude of the associations were stronger for MCS than for PCS, and bullying that started in adulthood or lasted > 24 months was more likely to be associated with this outcome. The MCS was at least four points lower among those who reported sexual abuse, independent of the age when it started, and was six points lower when this form of abuse occurred for 1 to 24 months.

**Table 3:** Adjusted association^a^ of bullying and sexual abuse with health-related quality of life (physical and mental component scores) among individuals ≥20 years in South Australia, 2015 (unweighted *N* = 2873)

		PCS	MCS
	%	β (95% CI)	β (95% CI)
BULLYING			
Age when started		*p* = 0.009^b^	*p* < 0.001^b^
Never	54.4	Ref	Ref
< 10 years	9.2	−1.2 (−2.5;0.0)	−1.7 (−2.9;-0.4)
10–19 years	20.7	−1.5 (−2.5;-0.4)	−1.5 (− 2.4;-0.6)
20+ years	15.7	− 1.1 (− 2.2;0.0)	−3.1 (−4.2;-2.1)
Duration (months)		*p* < 0.001^c^	*p* < 0.001^c^
Never	54.4	Ref	Ref
< 1 month	15.2	−0.9 (−1.9;0.2)	− 1.0 (−2.0;0.0)
1–24 months	23.2	− 1.3 (− 2.3;-0.3)	−2.5 (− 3.5;-1.6)
> 24 months	7.2	− 2.3 (− 3.7;-0.8)	− 3.1 (− 4.7;-1.5)
SEXUAL ABUSE			
Age when started		*p* = 0.006^b^	*p* < 0.001^b^
Never	89.6	Ref	Ref
< 10 years	3.4	−2.2 (−4.8;0.5)	−4.3 (−6.3;-2.4)
10–19 years	4.7	− 2.1 (−3.7;-0.5)	−4.6 (−6.7–2.6)
20+ years	2.4	−4.1 (−7.2;-1.0)	−4.1 (− 6.5;-1.7)
Duration (months)		*p* = 0.001^c^	*p* < 0.001^c^
Never	89.6	Ref	Ref
< 1 month	5.9	−2.1 (−3.9;-0.4)	−3.8 (−5.5;-2.1)
1–24 months	2.9	− 2.6 (−4.9;-0.4)	−6.4 (−8.6;-4.1)
> 24 months	1.6	− 3.9 (− 7.3;-0.5)	−3.1 (− 5.5;-0.6)

*PCS* physical component summary, *MCS* mental component summary, β = regression coefficient; *95% CI* 95% confidence interval

^a^Results adjusted for sex, age, marital status, area of residence, educational level, working status, socioeconomic position (Socio-Economic Indexes for Areas Index of Relative Socio-economic Advantage and Disadvantaged), and type of dwelling. Bullying and sexual abuse were also mutually adjusted

^b^Wald test for heterogeneity

^c^Wald test for trend

The magnitude and direction of the associations remained relatively stable when the results reported above were adjusted for mental health status (Additional files 3 and 4).

**Fig. 1:**
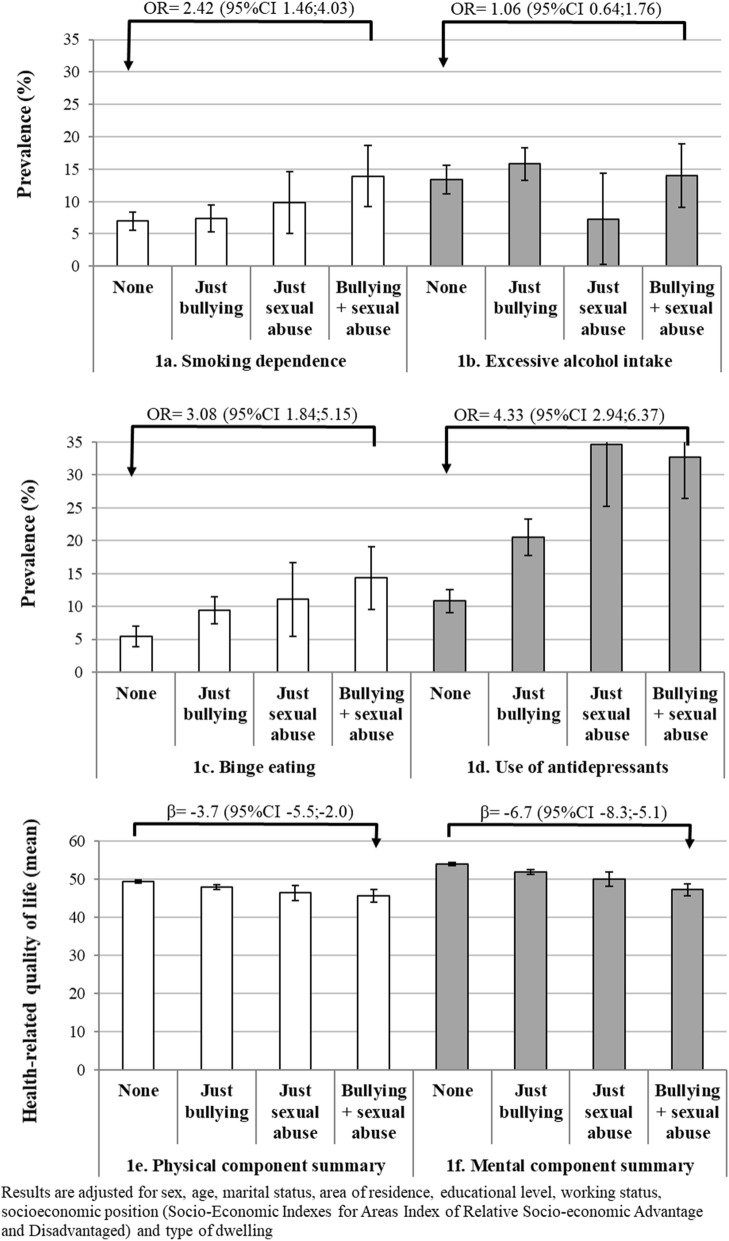
Adjusted combined effect of bullying and sexual abuse on smoking dependence, excessive alcohol intake, binge eating, antidepressant use and health-related quality of life among individuals ≥ 20 years in South Australia, 2015 (unweighted *N* = 2873). Vertical lines at the top of the columns represent the 95% CI for the respective prevalence or mean. β = regression coefficient; OR = odds ratio

When we investigated the association of bullying and sexual abuse combined (just bullying = 38.2%; just sexual abuse = 3.1%; both conditions = 7.3%) with these outcomes (Fig. 1), there was a cumulative adverse relationship with smoking dependence (Fig. 1a), binge eating (Fig. 1c), PCS (Fig. 1e) and MCS (Fig. 1f), with those who experienced both conditions showing the worst outcomes. The use of antidepressants was four times more frequent among individuals who reported sexual abuse (with or without bullying), while intermediate values were observed among those who experienced bullying but no sexual abuse (Fig. 1d). No association was observed with excessive alcohol intake (Fig. 1b).

**Fig. 2:**
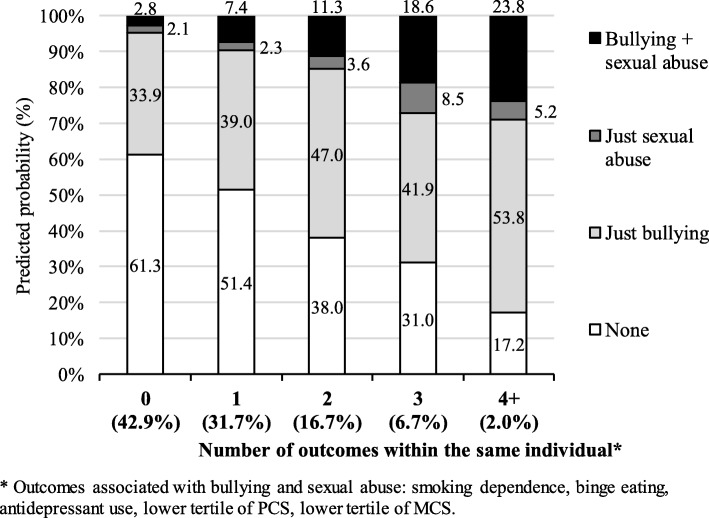
Predicted prevalence of bullying and sexual abuse according to the number of outcomes associated with these variables among individuals ≥ 20 years in South Australia, 2015 (unweighted N = 2873). PCS – physical component summary; MCS – mental component summary. The results were adjusted for sex, age, marital status, area of residence, educational level, working status, socioeconomic position (Socio-Economic Indexes for Areas Index of Relative Socio-economic Advantage and Disadvantaged), and type of dwelling

Based on these results, we predicted the probability of an individual having experienced bullying and/or sexual abuse based on his or her number of negative health outcomes (smoking dependence, binge eating, current use of antidepressants, lower PCS, and lower MCS) (Fig. 2). The likelihood of having suffered some form of abuse increased from 49.7% among those who had none of these outcomes to 82.8% among those with four or more negative health-related outcomes. The greatest increase was for the probability of having experienced both bullying and sexual abuse, which was eight times more likely among those who reported four or more outcomes.

None of the previously tested associations differed by sex or the use of medications for mental health problems (*p*-value for heterogeneity > 0.10 in all cases).

## Discussion

Five key findings of this population-based cross-sectional study can be highlighted. First, almost half of the adults of both sexes self-reported that they had been bullied, while nearly two out of ten women self-reported having ever been sexually abused, compared to one in thirty men. Second, one-third of the cases of bullying and 22.5% of the cases of sexual abuse started in adulthood. Independent of the age at onset, both forms of abuse were, in general, more likely to be associated with impaired mental health than with poor physical health. Third, 15% of individuals suffered these forms of abuse for more than 24 months, and except for alcohol intake and binge eating, long-lasting abuse showed a consistent adverse relationship with all other outcomes, including PCS. Fourth, bullying and sexual abuse showed a cumulative adverse association with smoking dependence, binge eating, and HRQoL (PCS or MCS), whereas sexual abuse was more likely than bullying to be related to antidepressant use. Finally, the likelihood of having ever been bullied or sexually abused was 83% among those with four or more outcomes (i.e., smoking dependence, binge eating, current use of antidepressants, lower PCS, and lower MCS), while the probability of having suffered both forms of abuse was 24%.

The prevalence of bullying in our sample was higher than the estimated 36% overall prevalence of school bullying [12–14] and the 17% prevalence of workplace bullying (considering random samples and the investigation of bullying using self-labelled questions without a definition) [33] reported in systematic reviews. However, a community-based Australian cohort study that used a single-item survey found a similar prevalence of bullying (46% of adults were “ever” bullied, and 7% were bullied at work in the previous six months) [34]. Estimates of bullying prevalence are influenced by the measurement method used (i.e., self-labelling vs a behavioural/operational approach), sampling procedures, and geographical differences [12–14, 33]. It has been suggested that self-labelling may underestimate the real prevalence of bullying compared to other approaches [35]. Nonetheless, an Australian study that included almost 1500 adults (48% males) found a very high concordance between self-labelling and a multi-dimensional scale of bullying behaviours (area under the curve 0.88) [34]. Therefore, the use of questions based on self-labelled bullying seems appropriate when investigating this form of abuse in large population-based surveys, especially when other health outcomes are investigated at the same time. However, although most cases of bullying are related to the school environment [12, 14], additional questions may be necessary to investigate other forms of bullying, such as cyberbullying or bullying in the workplace. In our study, one-third of all cases of bullying started in adulthood (15% among those aged 20–34 years, 44% at the ages of 35–64 years, 40% in the elderly; data not shown in tables), suggesting they are related to bullying outside of the school environment.

Similar methodological limitations apply to the investigation of sexual abuse, as the estimated frequencies vary according to the data source, the definition and severity of sexual abuse, and the investigated samples [8, 9, 36, 37]. However, our findings are consistent with the available literature. For example, in Australia, it has been estimated that the prevalence of childhood sexual abuse ranges from 11.6 to 21.5% in women and 4.1 to 7.5% in men (figures similar to those observed in other high-income countries) [21, 36]. Moreover, our results indicate that almost a quarter of the reported sexual abuse started in adulthood. This is consistent with a report by the World Health Organisation [8], which identified that up to 23% of adult women in high-income countries have ever been sexually assaulted by an intimate partner (6% in the previous 12 months). Therefore, although face-to-face interviews may affect the exploration of sensitive issues, such as sexual abuse, the consistency of our results with previous studies suggest the methodology used was able to provide valid estimates for this form of abuse. In fact, only 33 participants (1.1% of the sample) refused to answer the questions on sexual abuse, suggesting that this methodology could be used in other routine and social surveys in Australia.

When we examined the relationships between reported abuse and outcomes, the identified associations between single and combined estimates of bullying and physical and mental health problems are in the expected direction compared to the results of more detailed and lengthy instruments used in other studies. In 2017, a meta-analysis of 37 high-quality studies showed that those who suffered multiple forms of abuse in childhood were at moderate risk (ORs of two to three) for smoking, heavy alcohol use, and poor self-rated health compared to those with no history of childhood abuse. Consistent with our findings for antidepressant use and MCS, that study also found stronger associations with poor mental health (ORs of more than three) than with physical health variables [6]. Moreover, sexual abuse is a well-recognised risk factor for the development of eating disorders [38, 39], and physical or emotional abuse in childhood is associated with a three-fold higher risk of eating disorders [7]. Although the specific effects of bullying on this health outcome have been much less studied, a longitudinal study of children and adolescents in the United States showed that bullying victims were at increased risk of anorexia and bulimia nervosa in early adulthood. These results persisted even after the previous psychiatric status (including a history of eating disorder symptoms) and family adversities were considered [40].

More prolonged periods of abuse have been found to be related to more harmful effects [5, 7, 13], which was also observed in our study for most outcomes. The few exceptions included binge eating and MCS (associated with sexual abuse), for which abuse experiences of shorter duration were also detrimental. This effect may occur because, depending on the severity of the abuse, psychological reactions are triggered as soon as the aggression starts, leading to intense and unpredictable emotional responses that may vary considerably [10]. Furthermore, abuse episodes tend to be recurrent and coexist with other forms of maltreatment [3, 4, 6, 7, 10, 21]. In our study, 71% of the individuals who reported sexual abuse also suffered bullying at some time in their life. These two forms of abuse showed an additive adverse relationship with smoking dependence, binge eating, reduced PCS and MCS, but sexual abuse had a stronger association with antidepressant use than bullying.

Our results also showed that people living without a partner, those with an educational level equivalent to certificate/diploma, and those who were not currently working had a higher prevalence of both bullying and sexual abuse. We believe that health professionals should be aware that in addition to these sociodemographic risk factors, the likelihood of having ever been bullied or sexually abused is 83% among individuals who report the combination of smoking dependence, binge eating, antidepressant use, and lower HRQoL. One in four patients may have suffered both bullying and sexual abuse. Therefore, asking adult patients about their history of addictive behaviours, antidepressant use or reduced quality of life could help health professionals identify victims of abuse. Delays in the identification of such abuse may increase the risk of more severe adverse outcomes, including suicidality, major depressive disorders, or even intergenerational effects [5, 6, 13, 15, 21].

The strengths of our study include the investigation of a population-based sample (well distributed in terms of sex, age, and socioeconomic groups), the use of a well-recognised instrument to assess HRQoL, and the collection of additional relevant data (age at onset and duration) for bullying and sexual abuse.

However, some limitations should be recognised. First and most important, the cross-sectional design does not allow evaluation of the temporality of any associations (i.e., whether the health outcomes coincided with or preceded the experience of victimisation, or whether the individual sought or received treatment). Second, retrospective and uncorroborated accounts of early childhood abuse are subject to biases and omissions, especially among adults who suffer from depressive or alcohol-use disorder [41]. Moreover, correlated misclassifications and residual confounding cannot be excluded as all measures were self-reported, bullying and sexual abuse were investigated through self-labelling questions, and the results were not adjusted for socioeconomic conditions at the time the abuse was experienced. However, it is not likely that these sources of bias affected our results as only 1% of the participants refused to answer questions regarding these forms of abuse. The findings are also consistent with the results from research studies that utilised longitudinal designs and much more detailed and lengthy instruments [3, 4, 6, 7, 10, 21]. Finally, other addictive behaviours that are more strongly related to childhood abuse were not investigated [6]; such behaviours include illicit substance abuse/misuse, risky sexual behaviours, gambling, interpersonal and self-directed violence, or other forms of eating disorders (e.g., purging). Depression per se was also not investigated, although current use of antidepressants indicates that individuals were assessed and managed by a physician after the symptoms of depression were verified.

## Conclusions

This study shows that the use of self-labelling questions to investigate sensitive aspects, such as bullying and sexual abuse, in a survey is feasible. Such questions can provide estimates of prevalence and associations that are consistent with findings from studies using more detailed instruments (i.e., behavioural/operational approaches). Consistent with the available literature, sexual abuse and bullying were related to harmful behaviours (smoking dependence and binge eating), antidepressant use, and reduced HRQoL (especially MCS). These associations were identified even when the bullying or sexual abuse started in adulthood, and although there may be a dose-response effect, even the experience of a short duration of abuse appears to be related to these outcomes. Bullying is also common among sexually abused individuals, and both forms of abuse have an additive effect on health outcomes. Strategies that aim to prevent these forms of abuse are important. Identifying survivors of both forms of abuse is important to provide support and reduce more severe mental and physical consequences in the future.

## Additional files

Additional file 1: **Figure S1.** Duration of bullying and sexual abuse according to the age of onset of these forms of abuse among those ever bullied or ever sexually abused. (DOC 42 kb)

Additional file 2: **Figure S2.** Prevalence of bullying and sexual abuse according to age and stratified by sex. (DOC 44 kb)

Additional file 3: **Table S1.** Adjusted association of bullying and sexual abuse with smoking, alcohol intake, binge eating and antidepressant use. (DOC 71 kb)

Additional file 4: **Table S2.** Adjusted association of bullying and sexual abuse with health-related quality of life (physical and mental component scores). (DOC 69 kb)

## Abbreviations

95% CI: Confidence intervals of 95%
HRQoL: Health-related quality of life
MCS: Mental component score
OR: Odds ratio
p25-p75: Interquartile range
PCS: Physical component score
SA: South Australia
SEIFA-IRSAD: Australian Socio-Economic Indexes for Areas Index of Relative Socio-economic Advantage and Disadvantage
SF-12v1: Medical Outcomes Study Short Form 12
β: Regression coefficients
