# Supplementary material for: Bullying and sexual abuse and their association with harmful behaviours, antidepressant use and health-related quality of life in adulthood: a population-based study in South Australia
Source: BMC Public Health. 2019 Jan 7;19:26. doi: 10.1186/s12889-018-6367-8 (PMC6323811; doi:10.1186/s12889-018-6367-8)
Supplement: Supplementary file 1 — Figure S1. Duration of bullying and sexual abuse according to the age of onset of these forms of abuse among those ever bullied or ever sexually abused. (DOC 42 kb) [file 12889_2018_6367_MOESM1_ESM.doc]

**Supplementary Fig. S1. Duration of bullying and sexual abuse according to the age of onset of these forms of abuse among those ever bullied (unweighted n=1,251) or ever sexually abused (unweighted n=354).** Results adjusted for age and sex.
