# Supplementary material for: Bullying and sexual abuse and their association with harmful behaviours, antidepressant use and health-related quality of life in adulthood: a population-based study in South Australia
Source: BMC Public Health. 2019 Jan 7;19:26. doi: 10.1186/s12889-018-6367-8 (PMC6323811; doi:10.1186/s12889-018-6367-8)
Supplement: Supplementary file 2 — Figure S2. Prevalence of bullying and sexual abuse according to age and stratified by sex. (DOC 44 kb) [file 12889_2018_6367_MOESM2_ESM.doc]

**Supplementary Fig. S2. Prevalence of bullying and sexual abuse according to age and stratified by sex among individuals ≥20 years in South Australia, 2015 (unweighted N=2,873).** Vertical lines at the top of the columns represent the 95%CI for the respective prevalence. P-values for the interaction between sex and age were 0.059 for bullying and 0.049 for sexual abuse.
