# Supplementary material for: Bullying and sexual abuse and their association with harmful behaviours, antidepressant use and health-related quality of life in adulthood: a population-based study in South Australia
Source: BMC Public Health. 2019 Jan 7;19:26. doi: 10.1186/s12889-018-6367-8 (PMC6323811; doi:10.1186/s12889-018-6367-8)
Supplement: Supplementary file 3 — Table S1. Adjusted association of bullying and sexual abuse with smoking, alcohol intake, binge eating and antidepressant use. (DOC 71 kb) [file 12889_2018_6367_MOESM3_ESM.doc]

**Supplementary Table S1. Adjusted association^a^ of bullying and sexual abuse with smoking, alcohol intake, binge eating and antidepressant use** among individuals ≥20 years in South Australia, 2015 (unweighted N=2,873)

|  | **%** | **Smoking dependence^b^**  **(7.8%)** | **Excessive alcohol intake^c^**  **(14.3%)** | **Binge eating^d^**  **(8.1%)** |
| --- | --- | --- | --- | --- |
|  |  | **OR (95%CI)** | **OR (95%CI)** | **OR (95%CI)** |
| **BULLYING** |  |  |  |  |
| **Age when started** |  | p=0.563* | p=0.179* | p=0.049* |
| Never | 54.4 | Ref | Ref | Ref |
| <10 years | 9.2 | 1.47 (0.81;2.68) | 1.62 (1.04;2.51) | 1.71 (0.95;3.11) |
| 10-19 years | 20.7 | 0.94 (0.62;1.42) | 1.22 (0.88;1.71) | 1.52 (1.03;2.24) |
| 20+ years | 15.7 | 1.11 (0.72;1.71) | 1.09 (0.71;1.67) | 1.73 (1.11;2.70) |
| **Duration (months)** |  | p=0.114** | p=0.009** | p=0.008** |
| Never | 54.4 | Ref | Ref | Ref |
| <1 month | 15.2 | 0.87 (0.48;1.58) | 1.06 (0.68;1.65) | 1.42 (0.87;2.32) |
| 1-24 months | 23.2 | 0.95 (0.63;1.45) | 1.34 (0.97;1.85) | 1.83 (1.23;2.71) |
| >24 months | 7.2 | 2.08 (1.15;3.74) | 1.59 (1.02;2.46) | 1.40 (0.77;2.52) |
| **SEXUAL ABUSE** |  |  |  |  |
| **Age when started** |  | p=0.029* | p=0.269* | p=0.017* |
| Never | 89.6 | Ref | Ref | Ref |
| <10 years | 3.4 | 2.26 (1.10;4.65) | 1.05 (0.52;2.09) | 1.46 (0.87;2.45) |
| 10-19 years | 4.7 | 1.31 (0.74;2.33) | 0.72 (0.37;1.42) | 1.03 (0.56;1.89) |
| 20+ years | 2.4 | 2.46 (1.12;5.43) | 0.39 (0.14;1.13) | 2.93 (1.47;5.85) |
| **Duration (months)** |  | p=0.004** | p=0.235** | p=0.128 |
| Never | 89.6 | Ref | Ref | Ref |
| <1 month | 5.9 | 1.33 (0.79;2.22) | 0.81 (0.43;1.54) | 1.77 (1.02;3.10) |
| 1-24 months | 2.9 | 2.48 (1.25;4.92) | 0.53 (0.20;1.46) | 1.27 (0.71;2.27) |
| >24 months | 1.6 | 2.99 (1.14;7.87) | 0.88 (0.23;3.30) | 1.18 (0.49;2.85) |

OR = odds ratio; 95%CI = 95% confidence interval; *Likelihood-ratio test for heterogeneity; ** Likelihood-ratio test for trend

a - Results adjusted for sex, age, marital status, area of residence, educational level, working status, socioeconomic position (Socio-Economic Indexes for Areas Index of Relative Socio-economic Advantage and Disadvantaged), type of dwelling, **AND CURRENT USE OF SOME MEDICATION FOR MENTAL HEALTH PROBLEMS (INCLUDING ANTIDEPRESSANTS).** Bullying and sexual abuse are also mutually adjusted,

b – Heaviness of smoking index ≥3 points; c - ≥5 standard drinks of alcohol/day; d – overeating or eating an unusual large amount of food in one go (“out of control”) + distress associated to this behaviour
