# Supplementary material for: Bullying and sexual abuse and their association with harmful behaviours, antidepressant use and health-related quality of life in adulthood: a population-based study in South Australia
Source: BMC Public Health. 2019 Jan 7;19:26. doi: 10.1186/s12889-018-6367-8 (PMC6323811; doi:10.1186/s12889-018-6367-8)
Supplement: Supplementary file 4 — Table S2. Adjusted association of bullying and sexual abuse with health-related quality of life (physical and mental component scores). (DOC 69 kb) [file 12889_2018_6367_MOESM4_ESM.doc]

**Supplementary Table S2. Adjusted association^a^ of bullying and sexual abuse with health-related** quality of life (physical and mental component scores) among individuals ≥20 years in South Australia, 2015 (unweighted N=2,873)

|  | **%** | **PCS** |  | **MCS** |
| --- | --- | --- | --- | --- |
|  |  | **β (95%CI)** |  | **β (95%CI)** |
| **BULLYING** |  |  |  |  |
| **Age when started** |  | p=0.045* |  | p<0.001* |
| Never | 54.4 | Ref |  | Ref |
| <10 years | 9.2 | -1.1 (-2.3;0.1) |  | -1.3 (-2.5;-0.1) |
| 10-19 years | 20.7 | -1.2 (-2.2;-0.2) |  | -0.9 (-1.8;-0.1) |
| 20+ years | 15.7 | -0.8 (-2.0;0.3) |  | -2.5 (-3.5;-1.5) |
| **Duration (months)** |  | p=0.003** |  | p<0.001** |
| Never | 54.4 | Ref |  | Ref |
| <1 month | 15.2 | -0.7 (-1.7;0.4) |  | -0.7 (-1.7;0.2) |
| 1-24 months | 23.2 | -1.0 (-2.0;-0.1) |  | -1.9 (-2.8;-1.0) |
| >24 months | 7.2 | -1.9 (-3.3;-0.4) |  | -2.0 (-3.5;-0.5) |
| **SEXUAL ABUSE** |  |  |  |  |
| **Age when started** |  | p=0.046* |  | p<0.001* |
| Never | 89.6 | Ref |  | Ref |
| <10 years | 3.4 | -1.4 (-3.9;1.2) |  | -2.6 (-4.6;-0.6) |
| 10-19 years | 4.7 | -1.7 (-3.3;-0.2) |  | -3.8 (-5.9;-1.8) |
| 20+ years | 2.4 | -3.5 (-6.7;-0.3) |  | -2.7 (-4.8;-0.7) |
| **Duration (months)** |  | p=0.009** |  | p<0.001** |
| Never | 89.6 | Ref |  | Ref |
| <1 month | 5.9 | -1.7 (-3.4;-0.1) |  | -2.9 (-4.5;-1.3) |
| 1-24 months | 2.9 | -2.0 (-4.3;0.3) |  | -4.9 (-7.3;-2.5) |
| >24 months | 1.6 | -3.0 (-6.5;0.6) |  | -1.1 (-3.6;1.4) |

PCS – physical component summary; MCS – mental component summary; β = regression coefficient; 95%CI = 95% confidence interval; *Wald test for heterogeneity; ** Wald test for trend

a - Results adjusted for sex, age, marital status, area of residence, educational level, working status, socioeconomic position (Socio-Economic Indexes for Areas Index of Relative Socio-economic Advantage and Disadvantaged), type of dwelling, AND CURRENT USE OF SOME MEDICATION FOR MENTAL HEALTH PROBLEMS (INCLUDING ANTIDEPRESSANTS). Bullying and sexual abuse are also mutually adjusted
